# Bioactive compounds, antioxidant and antimicrobial activities of extracts from different plant parts of two *Ziziphus* Mill. species

**DOI:** 10.1371/journal.pone.0232599

**Published:** 2020-05-19

**Authors:** Yassine Yahia, Mohamed Ali Benabderrahim, Nizar Tlili, Mohamed Bagues, Kameleddine Nagaz

**Affiliations:** 1 Laboratoire d’Aridoculture et cultures oasiennes, Institut des Régions Arides, Médenine, Tunisia; 2 Département de Biologie, Faculté des Sciences de Tunis, Université Tunis El-Manar, Tunis, Tunisia; 3 Institut Supérieur des Sciences et Technologies de l’Environnement, Université de Carthage, Carthage, Tunisia; Institute for Biological Research, SERBIA

## Abstract

*Ziziphus lotus* L. (Lam.) and *Z*. *mauritiana* Lam., as a widespread species in Tunisia, are well known for their medicinal and food uses. The aim of the present study was to screen the content of total polyphenols, flavonoids, and condensed tannins together with the radical scavenging capacity and the antimicrobial activity of leaves, fruits and seeds extracts of *Z*. *lotus* and *Z*. *mauritiana* from different localities. Results showed that leaves extracts presented the highest phenolic compounds content for both species. Furthermore, LC-ESI-MS analysis allowed the identification of 28 bioactive compounds regardless of species and organs, with the predominance of quinic acid and rutin. Leaves extract of *Z*. *mauritiana* possessed the highest total antioxidant capacity. The antimicrobial tests showed that leaves extracts of *Z*. *mauritiana* and *Z*. *lotus* from Oued Esseder exhibited the highest activity against four bacterial strains (*Staphylococcus aureus*, *Listeria monocytogenes*, *Salmonella typhimurium* and *Escherichia coli*). The main results showed that the studied species of *Ziziphus* genus are an excellent source of natural bioactive molecules that could be an interesting material for industrial and food purposes.

## 1. Introduction

*Ziziphus* genus (Rhamnaceae family) comprises around 100 species [1], distributed in the tropical and sub-tropical regions across the world [2], which consist of trees, shrubs, climbers, and one herb [3]. It is known by the ancient Greeks as the tree zizyphon and in the Arabic called "zizouf", with reference to its mythical name [4]. The Rhamnaceae are regarded to be multipurpose plants and used as foods, folk medicines and environmental protector [5].

In Tunisia, three species are known: *Z*. *lotus*, *Z*. *spina-christi* and *Z*. *jujuba* [6]. *Z*. *lotus* grows mainly in the semi-arid and arid climate of Mediterranean, particularly in the Central and the South of Tunisia and it contributes to the landscape formation of these regions [7]. *Z*. *jujuba* native to India [8], occurs naturally in the south of Tunisia (Gafsa, Kebili and Sfax), while some trees are found in private gardens in the North regions (Ariana, Choutrana) [6]. *Z*. *mauritiana* or jujube of tropical Africa is widely known either as a spontaneous or naturalized shrub, or as a cultivated fruit tree that has become subspontaneous in some places [9]. It is a spiny, evergreen shrub or small tree up to 10 m in height, with a trunk of 30 cm or more in diameter and covered with a marked dark gray or dull black bark of irregular cracks; spreading crown; stipulated spines and several drooping branches. The shrub is commonly compact with only 3 to 4 m high, when the climatic conditions are extreme. The flowers are protandrous. Fruit set depends on insect cross-pollination attracted by the fragrance and nectar, and their development takes from 4 to 6 months of early and late cultivars, respectively [10]. This species is undoubtedly native to Africa and covers the most extensive area of the globe surface of all species of the genus *Ziziphus*. It occupies a huge area in Africa and constitutes the often dominant species of an association (climax) which is characteristic of a vast territory called "Sahelian or coastal zone", which extends in the South of the Sahara, from the Atlantic (Senegal and Mauritania) up to Somalia and extends even to Arabia and India regions. It is perfectly adapted to the climate of this area [9]. Indeed, *Z*. *mauritiana* is a hardy tree that thrives under fairly dry climate and copes with extreme temperatures, and its best fruit quality is closely related to hot, sunny and dry conditions [10].

Overall, various functional compounds such as vitamin C, amino acids, triterpene acids, polysaccharides, and polyphenols were previously reported in the *Ziziphus* genus [11, 12]. Further, works on this genus allowed the isolation and identification of flavonoids [13, 14], triterpene acids and derivatives such as saponins [15], alkaloids [16], indole derivatives [17], and fatty acids [5, 18]. In fact, saponins, flavonoid C-glycosides and fatty acids in some of the species of the *Ziziphus* were responsible of plants sedative and hypnotic effects [19]. Previous studies have reported that they possess multiple and specific activities, such as antidepressant-like [20], learning improvement and memory enhancement effects [21]. The contribution of amino acids and nucleosides of jujube in the regulation and modulation of various physiological processes in humans was recently reported by Guo et *al*. [22]. Traditionally, species belonging to the genus *Ziziphus* have been largely used as medicine to treat many diseases and body disorders, such as chest and respiratory problems, the scabies, the pimples, and the inflammation of mouth and gums. Furthermore, it has been reported that leaves of the *Ziziphus* species are efficient for bleaching face and neck, and treating hair growth [23]. In China, flowers of *Z*. *jujuba* Mill. were recommended as high-quality honey source and leaves were boiled and consumed as tea [24]. Likewise, fruit of *Z*. *mauritiana* is eaten fresh or dried and can be used as a condiment in a flour meal, butter, or cheese-like paste. It is an excellent source of carotene, fatty acids and vitamins A and C. Leaves are cooked as a vegetable or used as nutritious ovine and caprine feed in Indonesia and North Africa regions, respectively [10].

Among the studied compounds, phenolic substances, that include phenylpropanoids, flavonoids, catechins, tannins, and lignans [25], could display an exceptional array of biochemical and pharmacological activities, as single compounds or complex phytochemical mixtures. Recent pharmacological results have also revealed that polysaccharides, flavonoids, triterpenic and betulinic acids are the main active ingredients within the genus *Ziziphus* contributing respectively to its immune-modulating and hematopoietic functions [26], antioxidative effect [27, 28], anti-inflammatory and anticancer activities [29], and beneficial effects on cardiovascular system [30].

Most of the information reported in the literature has been restricted to *Z*. *lotus*, *Z*. *spina-christi* and *Z*. *jujuba*, while *Z*. *mauritiana* genotypes still remain under researched. This is mainly because the common consumer has no information about its health promoting effects and it was introduced and acclimatized in limited regions southern the country. Tunisia has remarkable biodiversity of the *Ziziphus* genus, and they are available including introduced, exotic and indigenous cultivars. Unfortunately, no systematic information is available on phytochemical composition and antioxidant activity of *Z*. *mauritiana* grown under Tunisian conditions.

To the best of our knowledge, there have been no reports on the effects of the species, environmental factors and organs on the antioxidant and antibacterial activities of the *Ziziphus* genus. Thus, the aim of the present study was to *i)* estimate the levels of total phenolic compounds and total flavonoids of leaves, fruits and seeds extracts of *Z*. *lotus* and *Z*. *mauritiana* L. from different localities *ii)* determine the qualitative and quantitative variation of phenolic acids and flavonoids using LC-ESI-MS, and *iii)* study the antioxidant and antibacterial activities.

## 2. Material and methods

### 2.1. Plant material

The plant material was collected from different regions of Médenine governorate (Southeast Tunisia), during July 2018. *Z*. *lotus* was collected from two localities; Bengardane (33°07'23" N/11°19'36" E) and Oued Esseder (33°19'13" N/10°40'33" E), however *Z*. *mauritiana* was provided by Arid Land Institute of Médenine (33°29'58" N/10°38'37" E). For each species, leaves, fruits and seeds were dried, powdered and finally stored in dark condition at -20 °C until phytochemical analyses.

### 2.2. Morphological traits

Morphological measurements were carried out at fruit ripening stage of the *Ziziphus* genus plants (summer 2018). The following measurements were taken on each plant: fruit length (mm), fruit width (mm), leaflet length (mm), leaflet width (mm), thorn length (mm), thorn spacing (mm), branch length (mm), 100 seeds weight (g), fruit color, leaflet color, and fruit taste.

### 2.3. Phenolic compounds analysis

#### 2.3.1. Sample preparation

The extraction of bioactive compounds was conducted according to the methods reported by Moore et *al*. [31], Abozed et *al*. [32], and Sarikurkcu et *al*. [33] with a slight modification. Samples were ground and the phenolic compounds were extracted using maceration in methanol (HPLC grade, ≥99.9%) at room temperature (25 °C). Briefly, 0.1 g of powder was dissolved in 10 ml of methanol (70%). The mixture was stirred for 24 hours and then centrifuged for 20 min at 5000 rpm. Finally, a Whatman paper (3 mm) was used to filter the mixture, and the obtained solutions were stored in the dark at 4 °C until further analysis.

#### 2.3.2. Total polyphenols content

Folin–Cicalteu reagent was used to determine the total phenolic compound concentrations as described by Singleton and Rossi [34]. Methanol extract (100 μl) was mixed with Folin reagent (750 μl) and 6% (w/v) sodium carbonate (750 μl). The absorbance was determined at 765 nm after incubation in the dark for 90 min at room temperature. The results were expressed as mg of gallic acid equivalents (GAE) per 100 g of dry weight (DW) (mg GAE/100 g DW).

#### 2.3.3. Total flavonoids content

According to the method described by Elfalleh et *al*. [35], the colorimetric assay was used to assess the total flavonoid content in all extracts. One ml of a fresh aluminum chloride solution (AlCl_3_, 2%) was added to 1 ml of suitably diluted samples extract/standard. The absorbance was determined at 460 nm, after incubation for 10 min at room temperature (25 °C). The total flavonoid content was expressed as mg quercetin equivalents (QE) per 100 g of dry weight (DW) (mg QE/100 g DW).

#### 2.3.4. Condensed tannins content

The condensed tannins content of different organ extracts was expressed as mg of catechin equivalents (CE) per 100 g of dry weight of plant material (mg CE/100 g DW). Vanillin solution (2 ml of 1% w/v vanillin in methanol) was mixed with sulfuric acid solution (2 ml of 25% v/v sulfuric acid in methanol) in the reaction tube. Then, 0.5 ml of suitably diluted samples was added to each tube. In the dark, the mixture was incubated and the absorbance was measured using a spectrophotometer (Perkin Elmer UV–VIS spectrophotometer) compared with the control tube at 500 nm after 15 min [36].

#### 2.3.5. Liquid chromatography-electrospray ionization-tandem mass spectrometry (LC-ESI–MS) analysis

Different organ extracts were dissolved in 1 ml of methanol (10%) and then the mixture was filtered through a membrane filter (0.45 μm) (Millipore, Billerica, MA, USA) and injected into the HPLC column. Separation of phenolic extracts was carried out by liquid chromatography-electrospray ionization-tandem mass spectrometry (LC–ESI–MS) analysis [37]. LC–ESI–MS analysis was performed adopting a LC–MS–2020 quadrupole mass spectrometer (Shimadzu, Kyoto, Japan) equipped with an electrospray ionization source (ESI) and managed in negative ionization mode. Mass spectrometer was coupled online with an ultra-fast liquid chromatography system including a binary pump system LC-20AD XR, DGU-20A 3R degasser, CTO-20AC column oven and auto-sampler SIL-20AC XR (Shimadzu, Kyoto, Japan). For analysis, Aquasil C18 guard (10 mm×3 mm, 3 μm) and Aquasil C18 columns (150 mm×3 mm, 3 μm) (Thermo Electron, Dreieich, Germany) were used. Both solvents A (0.1% formic acid in H_2_O, v/v) and B (0.1% formic acid in methanol, v/v) consisted of the mobile phase using a linear gradient elution: 0–45 min, 10–100% B; 45–55 min, 100% B. Between individual runs, the equilibrate period was controlled for five min. The injection volume and the flow rate of the mobile phase were 5 μL and 0.4 ml/min respectively and the temperature of the column was fixed at 40 °C. SIM mode (Selected Ion Monitoring) was used as spectra and treated using Shimadzu Lab Solutions LC–MS software. The mass spectrometer was operated with 3.5 kV capillary voltage, 1.5 L/min nebulizing gas flow, 12 L/min dry gas flow rate, 250 CDL (dissolving line), 400 °C block source temperature, 1.2 V voltage detector and 50 to 2000 Da full scan spectrum, in negative ion mode. Identification of the phenolic compounds present in different samples was achieved by comparison with retention times and spectra with 34 standard compounds (Sigma–Aldrich Company) based on external standard method by employing calibration curves in the range of 10–1000 μg/L.

### 2.4. Antioxidant activities

#### 2.4.1. Total antioxidant capacity (TAC)

The total antioxidant capacity of *Ziziphus* samples extracts was evaluated according to the method used by Tlili et *al*. [38]. First, a mixture of 0.1 ml of extract and 1 ml of reagent solution (0.6 M sulphuric acid, 28 mM sodium phosphate and 4 mM ammonium molybdate) was prepared. Then, the absorbance was measured at 695 nm after incubation for 90 min in boiling water. The total antioxidant capacity was expressed as mg of gallic acid equivalents per gram of dry weight (mg GAE/g DW).

#### 2.4.2. Free radical scavenging activity on (1, 1-diphenyl-2-picrylhydrazyl) (DPPH)

The DPPH radical scavenging assay was determined according to the method described by Bersuder et *al*. [39]. *Ziziphus* samples were diluted in methanol at different concentrations from 5 to 500 μg/ml. A quantity of 1 ml of each diluted fraction or extract was added to 250 μl of 0.2 nM DPPH methanolic solution. The mixture was stored in the dark for 45 min at room temperature (25 °C) with stirring. Finally, the DPPH reduction mixture was measured and compared to the DPPH methanolic solution or control at 517 nm. The DPPH radical scavenging capacity (%) was calculated as follows:

$$\text{DPPH}\;\text{radicals}\;\text{scavenged}\;\left(\%\right)=[({\text{A}}_{0}-{\text{A}}_{1})/{\text{A}}_{0}]\times 100$$

A_0_: the absorbance of the control reaction; A_1_: the absorbance of the tested extract sample.

DPPH scavenging activity is presented by IC_50_ value, defined as the concentration of the sample required to scavenge 50% of free radicals present in the test solution. Ascorbic acid was used as reference compounds.

### 2.5. Antimirobial activity

Standard and isolated strains of the following four bacteria, including two Gram-positive (*Staphylococcus aureus* (ATCC25923) and *Listeria monocytogenes* (ATCC19115)) and two Gram-negative (*Salmonella typhimurium* (NRLB4420) and *Escherichia coli* (ATCC35218)) bacteria were used in screening the antimicrobial activity of *Ziziphus* leaves extracts. The microorganisms were stored on Mueller–Hinton Agar (Bio-Rad) at 4 °C. In fact, microorganisms were obtained from the culture collection of the Arid Lands Institute of Médenine, Tunisia. The nutrient broth (Bio-Rad) and the Mueller–Hinton agar were used, respectively, for growing and diluting the microorganism suspensions for the antimicrobial assays. The paper disk agar diffusion was used to assess the antibacterial activities of leaves extracts according to the method described by Freney et *al*. [40]. Inoculates grown in the nutrient broth at 37 °C for 24 h were diluted to approximately 2 × 106 CFU/ml in molten nutrient agar. The final standard concentration of the suspension (used for inoculation) was obtained by adjusting the optical density to 0.5 at 570 nm wavelength (spectrophotometer UV/visible, Jenway 6405). Absorbent disks (Whatman disk No. 3 of 6-mm diameter) were impregnated with 20 μl of different extracts. Then, the disks were placed and incubated on the surface of inoculated plates (90 mm) at 37 °C for 24 h, respectively. Negative controls were prepared using a disk impregnated with the same solvent (methanol 70%) as that used to dissolve the plant extracts. Antimicrobial activity was evaluated by assessing the growth inhibition zone around the wells (diameter expressed in millimeters). This was the diameter of the zone visibly showing the presence or the absence of growth, including the 6-mm disk. All the tests were performed in triplicate.

### 2.6. Statistical analysis

All test trials were carried out in triplicate. Statistical data analyses of each plant organ (leaves, fruits and seeds) were computed using Xlstat 2018 software (www.xlstat.com). Results were calculated and expressed as mean values and standard deviations (mean ± SD). Multiple comparison tests (Post-hoc) were used, and differences among plant parts were analyzed based on Duncan's test at the p < 0.05 level.

## 3. Results and discussion

### 3.1. Morphological characterization of *Ziziphus* Mill. species

The quantitative traits carried out on the morphological data showed a significant variation of descriptors among species of the *Ziziphus* genus (*p*<0.05). *Z*. *mauritiana* showed the highest values for all characters, except for the thorns and branch length (Table 1). Qualitatively, the darkest color of leaves and fruits was observed in *Z*. *lotus*, respectively in Bengardane and Oued Esseder provenances (S1 Fig). The observed differences between these two species from different locations were similar to those reported in previous studies for *Z*. *lotus* [41] and Z. *mauritiana* [42, 43] and the set of *Ziziphus* genus [44, 45, 46]. It was clear that the interspecific differences of morphological traits among *Z*. *lotus* and *Z*. *mauritiana* in this study are very marked and allowed an easy species recognition. The significant intraspecific variability within *Z*. *lotus* samples could be attributed to environmental effects and depending on the sampling location [45, 47].

**Table 1:** Variability of the morphological parameters measured for *Ziziphus* Mill. species.

	*Ziziphus lotus*		*Ziziphus mauritiana*
	Bengardane	Oued Esseder	El Fjé
**Quantitative traits**			
Fruit length (mm)	12.19 ± 0.57^b^	12.98 ± 0.35^b^	16.19 ± 0.68^a^
Fruit width (mm)	11.21 ± 0.02^c^	11.42 ± 0.09^b^	11.63 ± 0.05^a^
Leaflet length (mm)	11.73 ± 0.07^c^	25.37 ± 0.71^b^	30.94 ± 0.56^a^
Leaflet width (mm)	10.66 ± 0.40^b^	15.53 ± 0.69^a^	15.99 ± 0.36^a^
Thorn length (mm)	11.69 ± 0.28^a^	12.51 ± 0.55^a^	06.05 ± 1.53^b^
Thorn spacing (mm)	16.36 ± 2.25^b^	18.16 ± 1.51^b^	24.29 ± 2.84^a^
Branch length (mm)	96.51 ± 6.63^b^	131.76 ± 22.69^a^	58.66 ± 8.59^c^
100 Seeds weight (g)	526.60 ± 2.08^c^	630 ± 36^b^	1396.60 ± 68^a^
**Qualitative traits**			
Fruit color	Rusted (dark)	Rusted (meduim)	Brown (light)
Leaflet color	Green (light)	Green (dark)	Green (medium)
Fruit taste	Sweet (+++)	Sweet (++)	Sweet (+)

Each value in the table is represented as mean ± SD (*n* = 3). Means in a column followed by the same letter are not significantly different by Duncan’s Multiple Range Test at the p *<* 0.05 levels

### 3.2. Total phenolic, flavonoid and condensed tannin contents

The profile of the secondary metabolites consists of a series of related compounds; that’s usually varied between plant organs, within developmental stages and sometimes even diurnally [48]. Among them, phenolic compounds are widely found in plants, which include a large group of bioactive ingredients [23]. Herein, for the studied species, the highest quantity of the total phenolic content was found in leaves; with values of 949.87 mg GAE/100 g DW in *Z*. *lotus* and 532.95 mg GAE/100 g DW in *Z*. *mauritiana*. However, the highest total phenolic compound in seeds (442.89 mg GAE/100 g DW) was determined in *Z*. *lotus* from Oued Esseder (Table 2). In the other hand, high values of total flavonoids were also detected in leaves of all samples with very similar pattern. Their concentrations were 92.22 and 91.89 mg QE/100 g DW in *Z*. *lotus* from Oued Esseder and Bengardane respectively, and 90.26 mg QE/100 g DW in *Z*. *mauritiana*. Also, results showed that the amount of total flavonoids differed significantly among organs of each sample. As regards the condensed tannins, the highest contents were detected in leaves of different *Ziziphus* samples and the most important amount was 216.21 mg CE/100 g DW in *Z*. *mauritiana* leaves from El Fjé.

**Table 2:** Total phenolic compounds, total flavonoids, condensed tannins contents and antioxidant activity of methanolic extracts of different *Ziziphus* Mill. species parts from Tunisia.

Species	*Ziziphus lotus*						*Ziziphus mauritiana*		
Origins	Bengardane			Oued Esseder			El Fjé		
Organs	Leaves	Fruits	Seeds	Leaves	Fruits	Seeds	Leaves	Fruits	Seeds
Total phenolic content (mg GAE/100 g DW*)	949.87 ± 28.37^a^	293.46 ± 17.20^e^	106.25 ± 16.65^h^	325.5 ± 10.13^d^	167.30 ± 7.10^f^	442.89 ± 34.94^c^	532.95 ± 17.03^b^	148.75 ± 5.35^g^	321.45 ± 21.47^de^
Flavonoids (mg QE/100 g DW**)	91.89 ± 5.23^a^	46.51 ± 1.95^b^	28.89 ± 8.01^cd^	92.22 ± 8.76^a^	21.21 ± 2.69^d^	84.54 ± 9.80^a^	90.26 ± 3.54^a^	39.33 ± 3.73^c^	41.29 ± 9.09^bc^
Condensed tannins (mg CE/100 g DW***)	89.08 ± 30.22^b^	32.03 ± 10.54^c^	26.02 ± 4.58^c^	82.08 ± 17.07^b^	37.03 ± 12.50^c^	30.03 ± 7.94^c^	216.21 ± 21.02^a^	26.02 ± 4.58^c^	70.07 ± 19.99^b^
TAC (mg GAE/g DW*)	30.95 ± 0.01^a^	23.87 ± 0.34b^c^	22.03 ± 3.08^c^	30.91 ± 0.06^a^	25.02 ± 0.55^b^	22.80 ± 0.15^bc^	31.01 ± 0.05^a^	21.76 ± 0.44^c^	21.63 ± 0.16^c^
**DPPH (IC_50_; μg/ml)**	18.27 ± 0.28^a^	12.16 ± 0.31^b^	18.57 ± 6.67^a^	16.60 ± 1.58^a^	15.15 ± 0.90^b^	11.41 ± 0.35^b^	18.80 ± 1.63^a^	14.07 ± 0.46^b^	12.62 ± 0.36^b^

* mg GAE/g DW: mg gallic acid equivalents per g dry residue;

** mg QE/g DW: mg quercetin equivalent per gram dry residue;

*** mg CE/g DW: mg catechin equivalent per gram dry residue. Each value in the table is represented as mean ± SD (*n* = 3). Means in a line followed by the same letter are not significantly different by Duncan’s Multiple Range Test at the p *<* 0.05 levels

These findings agree with previous studies reporting that *Ziziphus* leaves extract showed the highest total polyphenols content using different solvents of extraction compared to other plant parts [14, 49, 50] as well as to other species [51, 52, 53]. In the plant cell level, the phenolic compounds are usually present in the vacuoles of colored tissues such as leaves and/or flower petals [54]. Furthermore, previous studies revealed that the flavonoids located in the epidermis and/or cuticula of the leaves, as the lipophilic flavonoids, were identified among the most effective phenolic compounds conferring plant resistance against a wide range of crop enemies [54, 55]. Overall, the highest amounts of flavonoids were found in tropical plants in high altitudes areas than temperate zone ones do and their biosynthesis activities are depends largely to an excess of light or UV radiation [54, 56]. Tannins are abundant in many different plant species (*Quercus* spp., *Castanea* spp., *Rhus typhina*, *Tellima grandiflora*, *etc*.) and precisely located in the leaves, bark and fruits providing protection against many plant pathogens.

### 3.3. Antioxidant activity of *Ziziphus* sample extracts

The results showed that *Ziziphus* leaves presented the highest total antioxidant capacity (30~31 mg GAE/g DW) compared with other plant organs. Furthermore, significant differences of the antioxidant activity were found between samples of *Ziziphus* from different locations (Table 2). *Z*. *mauritiana* leaves with 31 mg GAE/g DW showed the highest total antioxidant capacity compared to those of Z. *lotus*, while Z. *mauritiana* seeds (21 mg GAE/g DW) showed the lowest value. These findings agree well with previous reports indicating that leaves of the *Ziziphus* genus showed a high antioxidant activity [14] due to their high bioactive compounds content such as polyphenols including tannins and flavonoids [57].

The DPPH through the IC_50_ values showed that fruits (12.16 μg/ml) and seeds (11.41 μg/ml) of Z. *lotus* from Bengardane and Oued Esseder were endowed with an interesting antioxidant activity. The antioxidant activity differences observed among different species of *Ziziphus* may be attributed to phenolic compounds, which depend on the region [23, 58], species [59], genotypes [60] and/or the extraction method [14]. These data extend to confirm the presence of substantial amounts of phenolics in Tunisian *Ziziphus* extracts indicating that they are a significant source of antioxidants which may provide health promoting advantages to the consumers.

### 3.4. Correlation analysis

To explore the *Ziziphus* bioactive molecules potential, correlation coefficients of the total phenolics, flavonoids and condensed tannins with the antioxidant capacity were analyzed (Table 3). It is common knowledge that the antioxidant activities are well correlated to the level of total phenolic compounds. Many researchers have reported that the most natural antioxidative molecules habitually work synergistically to create an antioxidative barrier against free radicals [14, 28, 57, 61]. Results of the current work clearly showed that the total flavonoid content (R^2^ = 0.853), condensed tannin content (R^2^ = 0.821) and then total phenolic content (R^2^ = 0.665) significantly correlated to total antioxidant activity. These findings indicated that flavonoids might be the most biologically active molecules of the phenolic compounds in the genus *Ziziphus*.

**Table 3:** Pearson correlations among phytochemical content and antioxidant potential of different *Ziziphus* Mill. species parts.

Variables	TPC	TFC	CTC
**TAC**	0.665*	0.853**	0.821**
**DPPH**	0.391	0.473	0.552

** Correlation is significant at the 0.01 level;

* Correlation is significant at the 0.05 level.

TPC: Total phenolic compound; TFC: Total flavonoids content;

CTC: Condensed tannins content; TAC: Total antioxidant capacity

Our findings appear to be well substantiated by Vermerris and Nicholson [54] reporting that the condensed tannins arise from polymerization of flavonoids, specifically flavan-3-ols, while hydrolyzable tannins are glycosylated gallic acids [62]. Furthermore, tannins are quite potent antibiotics [63].

### 3.5. Individual phenolic compounds

Twenty-eight phenolic compounds with diverse chemical structures were identified (Table 4). LC-ESI–MS analysis of the studied species showed that leaves extracts contained the highest amounts of phenolic compounds. *Z*. *mauritiana* leaves extract with nineteen identified molecules presented the largest number of individual phenolic compounds. The number of phenolic compounds detected in the present study is higher than those previously detected in *Ziziphus* genus [50, 60, 64]. Among the identified phenolics, quinic acid (65.12–902.4 μg/g), *p*-coumaric acid (2.58–15.67 μg/g) and rutin (9.29–4803.82 μg/g) were the only compounds shared between the different plant parts. Quinic acid serves as a precursor of shikimic acid and other secondary compounds synthesis [65] such as 4-O-caffeoylquinic acid, 1,3-di-O-caffeoylquinic acid, 3,4-di-O-caffeoylquinic acid and 4,5-di-O-caffeoylquinic acid, and a versatile chiral starting material for the synthesis of new pharmaceuticals [66]. Previous studies have reported lower amount of rutin in *Ziziphus* extracts [60, 67], while it is the most abundant flavonoid compound in the current study. The same results have been reported for some other popular species, such as asparagus spears [68], banana [69] and tomatoes [70]. The presence of *p*-coumaric acid, conjugate of rutin, was expected and has been previously reported [71]. According to Chen et *al*. [72], rutin and quercetin, as well as flavonoid mixtures of *Ginkgo biloba*, have been demonstrated as protectors of cerebellar cells from oxidative damage and apoptosis by scavenging hydroxyl radicals.

**Table 4:** Main detected compounds (μg/g DW) by LC-ESI-MS from different parts of *Ziziphus* Mill. species in Tunisia.

Samples	*Ziziphus lotus*						*Ziziphus mauritiana*		
	Bengardane			Oued Esseder			El Fjé		
Compounds	Leaves	Fruits	Seeds	Leaves	Fruits	Seeds	Leaves	Fruits	Seeds
Quinic acid	108.86 ± 11.58f^g^	162.02 ± 9.29^e^	65.12 ± 8.34^h^	418.12 ± 36.89^c^	149.02 ± 25.32e^f^	88.10 ± 14.19^gh^	800.01 ± 46.26^b^	902.40 ± 31.70^a^	258.94 ± 17.71^d^
Gallic acid	-	-	5.59 ± 0.29^b^	10.47 ± 1.79^a^	-	4.37 ± 0.86^b^	10.36 ± 1.79^a^	-	-
Catechin (+)	3.31 ± 0.33^c^	-	2.18 ± 0.09^d^	7.71 ± 2.32^b^	-	1.56 ± 0.29^e^	296.01 ± 14.68^a^	-	1.29 ± 0.10^e^
Chlorogenic acid	-	7.58 ± 0.22^b^	10.59 ± 0.68^a^	-	7.28 ± 0.38^b^	9.88 ± 0.05^a^	-	-	-
4-O-caffeoylquinic acid	0.31 ± 0.04^c^	-	-	-	-	1.16 ± 0.14^b^	23.34 ± 6.41^a^	-	0.24 ± 0.03^c^
Caffeic acid	-	-	-	-	-	-	4.95 ± 1.52^a^	-	-
Syringic acid	-	-	0.58 ± 0.14^b^	1.96 ± 0.31^a^	-	-	0.41 ± 0.11^b^	-	-
1,3-di-O-caffeoylquinic acid	-	-	-	-	1.38 ± 0.21^a^	-	-	-	-
Epicatechin	-	2.20 ± 0.28^b^	-	2.45 ± 0.19^b^	-	-	46.49 ± 5.51^a^	-	-
*P*-coumaric acid	3.30 ± 0.21^d^	5.01 ± 0.09^c^	2.58 ± 0.53^d^	6.53 ± 0.58^b^	3.22 ± 0.25^d^	2.62± 0.25^d^	15.67 ± 2.91^a^	7.06 ± 0.96^b^	5.19 ± 0.66^bc^
Trans ferulic acid	-	-	-	0.99 ± 0.09^c^	-	-	4.20 ± 0.83^a^	1.69 ± 0.16^b^	1.45 ± 0.04^b^
Hyperoside	-	-	1.75 ± 0.42^c^	375.57 ± 10.11^b^	-	1.91 ± 0.45^c^	1226.51 ± 225.88^a^	-	-
Rutin	2921.52 ± 11.49^b^	82.85 ± 11.88^d^	12.49 ± 1.33^f^	4803.82 ± 468.69^a^	162.27 ± 3.41^c^	14.63 ± 0.55^f^	3290.10 ± 396.82^b^	34.99 ± 0.11^e^	9.29 ± 0.12^g^
Luteolin-7-o-glucoside	-	-	-	-	1.32 ± 0.01^a^	-	-	-	-
3,4-di-O-caffeoylquinic acid	-	-	-	-	-	5.71 ± 0.44^a^	-	-	-
Quercitrin	17.43 ± 0.13^b^	5.7 ± 0.07^e^	-	53.98 ± 16.59^a^	6.09 ± 0.03^c^	5.84 ± 0.10^de^	93.45 ± 28.76^a^	5.92 ±0.02^d^	-
Naringin	10.94 ± 0.40^b^	0.87 ± 0.13^c^	0.95 ± 0.01^c^	37.68 ± 8.26^a^	-	-	-	-	-
Apigenin-7-o-glucoside	-	0.13 ± 0.01^c^	0.49 ± 0.05^a^	-	0.09 ± 0.01^c^	0.27 ± 0.04^b^	-	-	0.09 ± 0.03^c^
4,5-di-O-caffeoylquinic acid	-	-	-	-	-	10.46 ± 1.10^a^	9.30 ± 0.70^a^	-	2.11 ± 0.26^b^
Salviolinic acid	-	-	-	-	-	-	10.58 ± 1.61^a^	-	-
Trans cinnamic acid	-	-	9.98 ± 0.63^b^	19.21 ± 1.39^a^	-	-	10.19 ± 2.02^b^	-	-
Quercetin	0.96 ± 0.01^d^	-	0.98 ± 0.10^d^	2.63 ± 0.19^b^	0.78 ± 0.02^e^	-	4.89 ± 0.97^a^	1.21 ± 0.01^c^	-
Kaempferol	-	-	-	2.13 ± 0.35^a^	-	-	-	-	-
Naringenin	-	-	0.46 ± 0.06^c^	0.62 ± 0.08^c^	-	2.94 ± 0.09^a^	1.12 ± 0.06^b^	1.24 ± 0.08^b^	1.33 ± 0.20^b^
Apigenin	-	-	0.26 ± 0.01^c^	0.35 ± 0.04^b^	-	0.79 ± 0.08^a^	-	-	-
Luteolin	2.31 ± 0.10^e^	-	8.33 ± 0.38^b^	2.85 ± 0.60^e^	-	43.65 ± 4.18^a^	4.98 ± 0.76^d^	-	6.21 ±0.01^c^
Cirsilineol	-	-	7.01 ± 0.24^b^	-	-	39.35 ± 2.86^a^	2.77 ± 0.83^d^	-	5.31 ± 0.08^c^
Acacetin	-	-	-	0.29 ± 0.13^a^	-	0.22 ± 0.02^a^	-	-	-

Results were given as means ± SD. Means in a line followed by the same letter are not significantly different by Duncan’s Multiple Range Test at the p *<* 0.05 levels; -: not detected.

In addition, other phenolic acids compounds such as trans ferulic acid, 3,4-di-O-caffeoylquinic acid (isochlorogenic acid C), 4,5-di-O-caffeoylquinic acid (isochlorogenic acid b), salviolinic acid and trans cinnamic acid and other flavonoids such as luteolin-7-o-glucoside, apigenin-7-o-glucoside, apegenin, luteolin, cirsilineol and acacetin were identified. In the literature, these natural compounds have not been previously detected in *Ziziphus* extracts. Tlili et *al*. [73] have identified similar compounds in *Periploca laevigata* extract and have described their considerable interest due to their industrial potential, pharmacological and medicinal values such as antioxidant, anti-inflammatory and analgesic proprieties.

From a geographical point of view, there were great variations in the phenolic composition between *Z*. *lotus* samples. Indeed, some compounds were exclusively detected in Oued Esseder plants (1,3-di-O-caffeoyquinic acid, trans ferulic acid, Luteolin-7-o-glucoside, 3,4-di-O-caffeoyquinic acid, 4,5-di-O-caffeoyquinic acid, kampherol and Acacetin) compared with samples collected from Bengardane. In plants, the differences of phenolic compounds accumulation between locations, notably with nutritional and/or medicinal properties have been previously described by Chirinos et *al*. [74]. Depending on specific/genetic variation, some phenolic compounds were particularly identified in Z. *lotus* (chlorogenic acid, 1,3-di-O-caffeoyquinic acid, Luteolin-7-o-glucoside, naringin, kampherol and acacetin) while others are unique to *Z*. *mauritiana* samples (caffeic and salviolinic acids).

Overall, the high levels of polyphenols, cafeic acid derivatives (4-O-caffeoylquinic acid, Caffeic acid, 1,3-di-O-caffeoylquinic acid, 3,4-di-O-caffeoylquinic acid, 4,5-di-O-caffeoylquinic acid) and other bioactive compounds (eg. chlorogenic, *P*-coumaric and quinic acids) have been previously detected in coffee and others species extracts (guayusa, mate, camellia and cocoa extracts) [75, 76]. These compound that given a stimulating or refreshing effect on the brain providing sedative and hypnotic activities by calming down the mind and improving quality of sleep, through consuming *Ziziphus* fruits [23]. Traditionally and for the same reason, *Ziziphus* fruits are similarly consumed in our region due to their health benefits, as both food and herbal medicine.

### 3.6. Antimicrobial activity

In order to survive, plants need to develop defense strategies against herbivores, microbes (bacteria, fungi), viruses and even against other plants competing for light, space and nutrients [25]. Plants are always a rich source of compounds that do not appear essential for primary metabolism, including thousands of secondary metabolites. In addition to their function in physiology or in structural maintenance, some of the secondary metabolites (e.g. flavonoids, anthocyanins, tetraterpenes and monoterpenes) serve for defense against microbes or herbivorous animals and as signal functions at the same time [25, 77]. As shown in Table 5, the antimicrobial activity of *Ziziphus* leaves extract was tested at 10 mg/ml against two Gram-positive (*Staphylococcus aureus* and *Listeria monocytogenes*) and two Gram-negative (*Salmonella typhimurium* and *Escherichia coli*) bacteria. The maximal inhibitions were observed against *S*. *aureus* with *Z*. *lotus* of Bengardane (13 ± 0.2 mm) and *Z*. *mauritiana* (14.2 ± 0.27 mm). *Ziziphus* leaves extract of El Fjé and Oued Esseder were found to be more effective against all bacterial strains.

**Table 5:** Antibacterial activity of *Ziziphus* leaves extracts at 10 mg/ml against Gram-positive and Gram-negative bacterial strains.

	*Ziziphus lotus*		*Ziziphus mauritiana*	Gentamicin Disks (30 μg)
Strains	Bengardane	Oued Esseder	El Fjé	
*S*. *aureus*	13 ± 0.2^b^	12.2 ± 0.11^c^	14.2 ± 0.27^a^	30
*L*. *monocytogenes*	10 ± 0.08^b^	12.2 ± 0.30^a^	12 ± 0.5^a^	26
*S*. *typhimurium*	12.2 ± 0.17^a^	11.2 ± 0.36^a^	11.4 ± 0.57^a^	20
*E*. *coli*	10.6 ± 0.16^b^	11.8 ± 0.64^a^	10.4 ± 0.45^b^	18

Data are expressed in diameter (in mm) of bacteria inhibition growth and given in mean ± SD; Different letters for the same line indicate significant differences (p < 0.05).

The antimicrobial potential of the extracts of *Ziziphus* genus leaves was supported by previous works. In this study, their inhibitory effect was higher than those reported by Elaloui et *al*. [59] and Naili et *al*. [78], whose revealed that they exhibited the highest antibacterial activity with a zone of inhibition ranging from 7 to 18 mm, and 10 to 15 mm respectively, against *S*. *aureus*, *S*. *typhimurium* and *E*. *coli*. In fact, the higher antimicrobial activity of leaves extract of El Fjé and Bengardane appeared to be principally due to their richness with bioactive compounds such as polyphenols, flavonoids and tannins, as shown previously. The obtained results were in accordance with previous findings [25] showing that the plant antimicrobial activity is mainly due to flavonoids, tannins, phenolic compounds, alkaloids and terpenoids.

The significant differences in the antimicrobial activity observed among *Z*. *lotus* samples, could be attributed to the variability in phenolic compounds, depending on the sampling location and environmental effects as previously reported for other species [52, 61, 79]. Of these phenolic compounds, 3,4-dihydroxyphenyl ethanol glucoside displays great bacteriostatic activity against *S*. *typhimurium*, *S*. *aureus*, *Bacillus aureus* and *E*. *coli* [80]. Also, the mutagenicity of quercetin has been established in many experiments using prokaryotic and eukaryotic organisms. According to Wink et *al*. [25], quercetin is the most active flavonoid that possibly acting via intercalation into DNA, inducing frameshift mutations. In the same way, Bernard et *al*. [81] described a DNA topoisomerase inhibitor specific for topoisomerase IV, which promoted *E*. *coli* topoisomerase IV-dependent DNA cleavage. They suggested that rutin is the most active compound that inhibits topoisomerase IV-dependent decatenation activity (concentration required to produce half maximum inhibition IC_50_ 64 μg/ml). Interestingly, as indicated by Wink et *al*. [25], the usefulness of bioactive plant-derived secondary metabolites leads to produce new and more active antimicrobial agents.

## Conclusion

In order to assess the bioactive compounds of two wild of the genus *Ziziphus* collected from Tunisian arid zones, an LC-ESI-MS analysis was used to realize a detailed profile of the phenolic compounds from three organs of *Z*. *lotus* and *Z*. *mauritiana*. The antioxidant activities and the antimicrobial potential against four pathogenic bacteria strains were evaluated. In total, twenty-eight phenolic compounds were identified and quantified in different extracts. The leaves were endowed with phenolic compounds (10 phenolic acids and 9 flavonoids) showing considerable antioxidant and antimicrobial properties compared to the other organs. The quinic acid, *p*-coumaric acid, rutin and quercitrin were the predominant compounds confirming the potential uses of both species as sources of bioactive molecules. Quantitatively, the observed differences among phytochemical concentrations of the sister species *Z*. *lotus* and *Z*. *mauritiana* are very marked and allowed easily to discriminate between species. Thus, further investigations should carry out for individual phenolic compounds for potential medicinal and industrial uses.

## Supporting information

S1 Fig Graphical abstract. (DOCX)

S2 Fig Morphological characterization of *Ziziphus* L. species. Morphological characterization of leaves and fruits in *Ziziphus* L. species (Bengardane (a), Oued Esseder (b) and El Fjé (c)) from Tunisia.
(DOC)

S3 Fig Representative chromatograms of phenolic compounds identified by LC-ESI-MS from different parts of *Ziziphus* Mill. species in Tunisia. (DOC)

S1 Table Characterization of phenolic compounds in the *Ziziphus* Mill. species by LC-MS using ESI negative ion mode. (DOC)
