# Supplementary figures and images for: Bioactive compounds, antioxidant and antimicrobial activities of extracts from different plant parts of two Ziziphus Mill. species
Source: PLoS One. 2020 May 19;15(5):e0232599. doi: 10.1371/journal.pone.0232599 (PMC7236975; doi:10.1371/journal.pone.0232599)

**S1 Fig. Graphical abstract.**

**Bioactive compounds from two *Ziziphus* Mill. species**


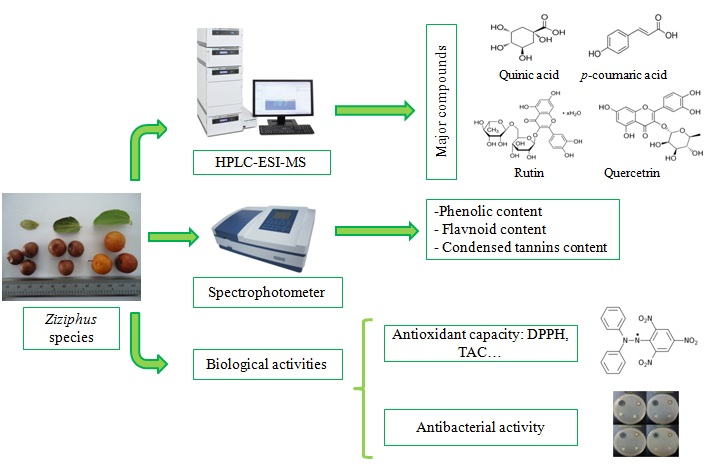

Supplement: S1 Fig — (DOCX) [file pone.0232599.s001.docx]
