# Supplementary material for: Bioactive compounds, antioxidant and antimicrobial activities of extracts from different plant parts of two Ziziphus Mill. species
Source: PLoS One. 2020 May 19;15(5):e0232599. doi: 10.1371/journal.pone.0232599 (PMC7236975; doi:10.1371/journal.pone.0232599)
Supplement: S2 Fig — Morphological characterization of leaves and fruits in Ziziphus L. species (Bengardane (a), Oued Esseder (b) and El Fjé (c)) from Tunisia. (DOC) [file pone.0232599.s002.doc]

**S1 Fig.**

**
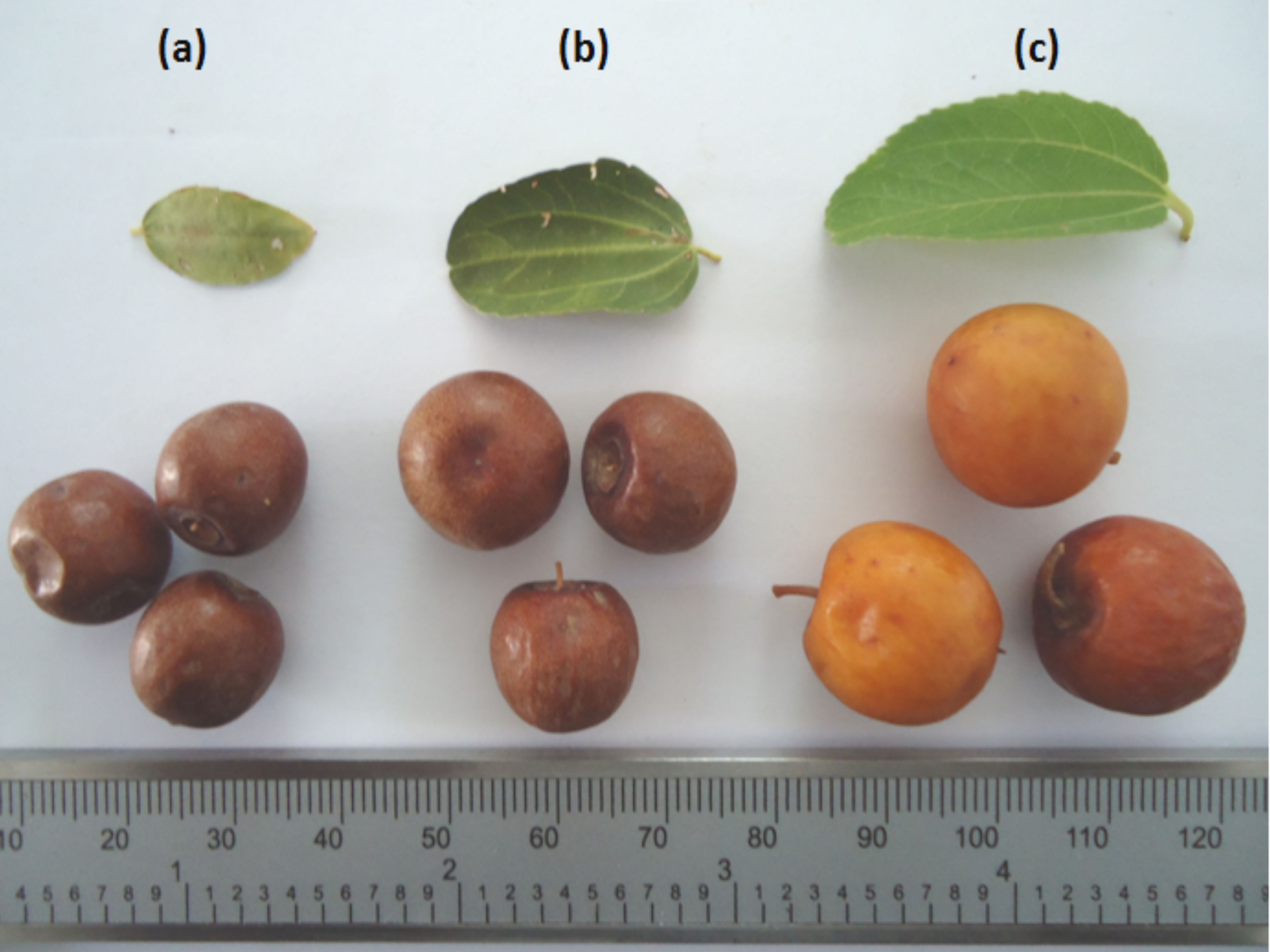
**

**Figure 1:** Morphological characterization of leaves and fruits in *Ziziphus* L. species (Bengardane (**a**), Oued Seder (**b**) and El Fjé (**c**)) from Tunisia.
