# Supplementary material for: Bioactive compounds, antioxidant and antimicrobial activities of extracts from different plant parts of two Ziziphus Mill. species
Source: PLoS One. 2020 May 19;15(5):e0232599. doi: 10.1371/journal.pone.0232599 (PMC7236975; doi:10.1371/journal.pone.0232599)
Supplement: S3 Fig — (DOC) [file pone.0232599.s003.doc]

**S3 Fig.** Representative chromatograms of phenolic compounds identified by LC-ESI-MS from different parts of *Ziziphus* Mill. species in Tunisia.


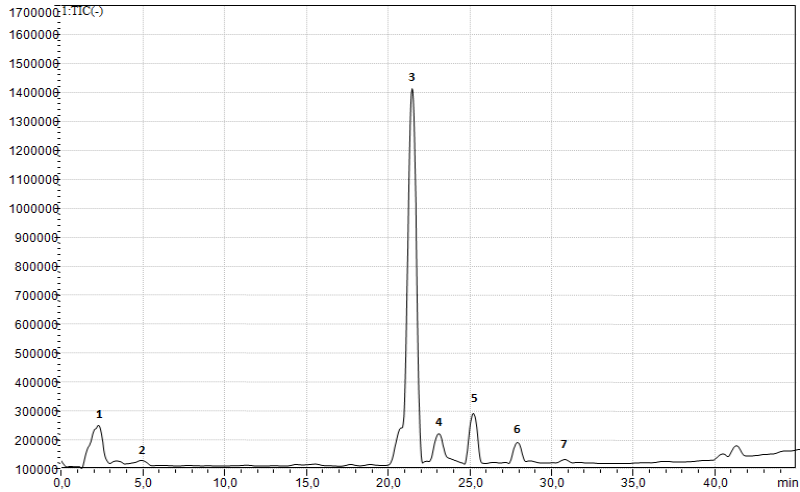


**Fig. 1.** Major detected phenolic compounds by LC-ESI-MS in *Ziziphus* *lotus* (Oued Esseder) leaves extracts; Peak numbers designate identified compounds: 1, 2, 3, 4, 5, 6, and 7; 1. Quinic acid 2. Gallic acid 3. Rutin 4. Hyperoside 5. Naringin 6. Quercitrin 7. Trans cinnamic acid


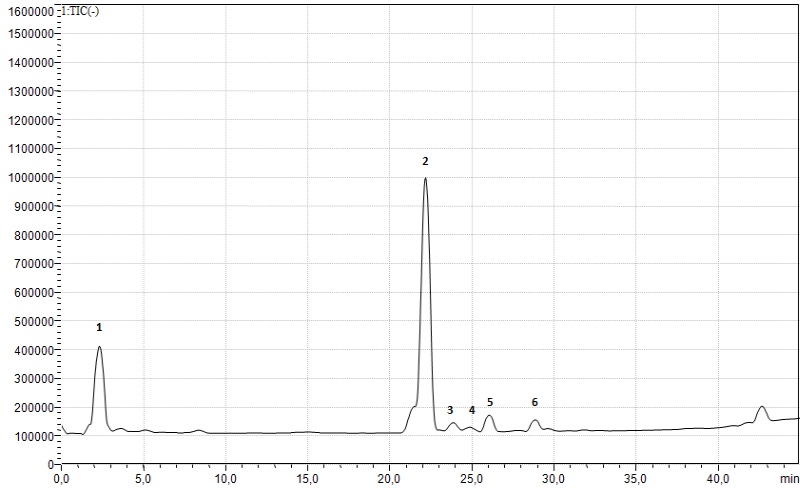


**Fig. 2.** Main detected phenolic compounds by LC-ESI-MS from *Ziziphus* *mauritiana* (El Fjé) leaves extracts; Peak numbers designate identified compounds: 1, 2, 3, 4, 5, and 6; 1. Quinic acid 2. Rutin 3. Hyperoside 4. Quercitrin 5. 4,5-di-O-caffeoylquinic acid 6. Salviolinic acid
