# Supplementary material for: Bioactive compounds, antioxidant and antimicrobial activities of extracts from different plant parts of two Ziziphus Mill. species
Source: PLoS One. 2020 May 19;15(5):e0232599. doi: 10.1371/journal.pone.0232599 (PMC7236975; doi:10.1371/journal.pone.0232599)
Supplement: S1 Table — (DOC) [file pone.0232599.s004.doc]

S1 Table.

Table 1. Characterization of phenolic compounds in the *Ziziphus* Mill. species by LC-MS using ESI negative ion mode.

| **Compounds** | **Molecular formula** | **[M-H]−m/z** | **Retention time (min)** |
| --- | --- | --- | --- |
| Quinic acid | C_7_H_12_O_6_ | 191.00 | 2.079 |
| Gallic acid | C_7_H_6_O_5_ | 169.00 | 3.980 |
| Catechin (+) | C_15_H_14_O_6_ | 289.00 | 11.096 |
| Chlorogenic acid | C_16_H_18_O_9_ | 353.00 | 9.232 |
| 4-O-caffeoylquinic acid | C_16_H_18_O_9_ | 353.00 | 11.604 |
| Caffeic acid | C_9_H_8_O_4_ | 179.00 | 14.466 |
| Syringic acid | C_9_H_10_O_5_ | 197.00 | 16.000 |
| 1,3-di-O-caffeoylquinic acid | C_25_H_24_O_12_ | 515.00 | 16.998 |
| Epicatechin | C_15_H_14_O_6_ | 289.00 | 16.273 |
| *P-*coumaric acid | C_9_H_8_O_3_ | 163.00 | 20.859 |
| Trans ferulic acid | C_10_H_10_O_4_ | 193.00 | 23.137 |
| Hyperoside | C_21_H_20_O_12_ | 463.00 | 24.451 |
| Rutin | C_27_H_30_O_16_ | 609.00 | 23.688 |
| Luteolin-7-o-glucoside | C_21_H_20_O_11_ | 447.00 | 24,181 |
| 3,4-di-O-caffeoylquinic acid | C_25_H_24_O_12_ | 515.00 | 24.839 |
| Quercitrin | C_21_H_20_O_11_ | 447.00 | 26.373 |
| Naringin | C_15_H_12_O_5_ | 579.00 | 25.807 |
| Apigenin-7-o-glucoside | C_21_H_20_O_10_ | 431.00 | 26.715 |
| 4,5-di-O-caffeoylquinic acid | C_25_H_24_O_12_ | 515.00 | 26.645 |
| Salviolinic acid | C_36_H_30_O_16_ | 717.00 | 27.999 |
| Trans cinnamic acid | C_9_H_8_O_2_ | 147.00 | 31.919 |
| Quercetin | C_15_H_10_O_7_ | 301.00 | 31.709 |
| kaempferol | C_15_H_10_O_6_ | 285.00 | 31.805 |
| Naringenin | C_15_H_12_O_5_ | 271.00 | 33.729 |
| Apigenin | C_15_H_10_O_5_ | 269.00 | 34.248 |
| Luteolin | C_15_H_10_O_6_ | 285.00 | 34,673 |
| Cirsilineol | C_17_H_14_O_7_ | 343.00 | 38.314 |
| Acacetin | C_16_H_12_O_5_ | 283.00 | 40.052 |
